# Enhanced anti-colorectal cancer effects of carfilzomib combined with CPT-11 via downregulation of nuclear factor-κB *in vitro* and *in vivo*

**DOI:** 10.3892/ijo.2014.2513

**Published:** 2014-06-23

**Authors:** WEIWEI TANG, GUANGJIAN SU, JIEYU LI, JINRONG LIAO, SHUPING CHEN, CHUANZHONG HUANG, FANG LIU, QIANG CHEN, YUNBIN YE

**Affiliations:** 1 Graduate School of Education, Fujian Medical University, Fuzhou, P.R. China; 2 Laboratory of Immuno-Oncology, Fujian Provincial Cancer Hospital, Fuzhou, P.R. China; 3 Department of Medical Oncology, Fujian Medical University Union Hospital, Fuzhou, P.R. China; 4 Fujian Provincial Key Laboratory of Translational Cancer Medicine, Fuzhou, P.R. China

**Keywords:** carfilzomib, irinotecan, synergistic, colorectal carcinoma, nuclear factor-κB

## Abstract

Upregulation of nuclear factor-κB (NF-κB) in colorectal carcinoma (CRC) accelerates tumor growth, whereas, irinotecan (CPT-11)-induced NF-κB activation reduces chemosensitivity and weakens the anti-colorectal cancer function itself, while proteasome inhibitors can inhibit NF-κB and improve the effect of chemotherapy. Carfilzomib (CFZ) is a novel proteasome inhibitor that has been recently approved by the FDA and is in clinical use for the treatment of multiple myeloma, but little is known about its activity against CRC. The aim of the present study was to explore whether CFZ alone or in combination with CPT-11 is effective in CRC treatment. We evaluated the novel therapeutic ability and mechanism of action of CFZ in CRC *in vitro* and *in vivo*. SW620 cells were incubated with CFZ alone or in combination with CPT-11. Cell proliferation was assessed by WST-1 and clonogenic assays, the cytotoxic interaction was assessed with a combination index (CI). Cell cycle progression was analysed with flow cytometry. Cell apoptosis was evaluated by detecting the Annexin V/propidium iodide (PI) ratio, caspase 3 and CD95 expression, and with TUNEL staining. Cell migration and invasion was determined with a wound-healing assay and a Transwell matrix penetration assay. A CRC xenograft model was established to monitor tumor growth. EMSA was used to analyse NF-κB activation and western blot analysis was used to detect the protein levels of related signaling factors. CFZ significantly inhibited the growth of SW620 cells, and had synergistic inhibitory effects with CPT-11 on survival and colony formation; possibly by inhibition of NF-κB activation, MEK/ERK and PI3K/AKT pathway factor dephosphorylation and survivin downregulation. Co-administration of CFZ and CPT-11 induced G2/M arrest, increased p21^WAF1/CIP^, and decreased mutant p53 and cdc25c expression. Induction of apoptosis was accompanied by marked increases in PARP cleavage, caspase 3 activation, an increase of CD95 and p-p38, and ATF3 activation. Combination treatment lowered the invasive and migration ability of SW620 cells, reduced MMP and increased TIMP protein expression. Finally, co-administration of CFZ and CPT-11 suppressed tumor growth and increased apoptosis compared with single-agent treatment in SW620 xenograft models correlated with NF-κB downregulation. Carfilzomib alone or in combination with CPT-11 is effective against colorectal cancer through inhibition of multiple mechanisms related to NF-κB, and could be a potential novel therapy for CRC.

## Introduction

Colorectal cancer is a leading malignancy worldwide with more than 1,233,000 new cases and 608,000 deaths in recent years (1). In current chemotherapeutic treatment for CRC, irinotecan (CPT-11), a semisynthetic derivative of camptothecin, is one of the key cytotoxic drugs with a 30% single agent response rate and 50% response rate when combined with other agents such as 5-Fu/leucovorin (LV) (2). However, more than 40% of patients treated with this drug have severe toxicities, such as diarrhea and neutropenia that limit its efficacy (3), and there remain many patients treated with CPT-11 who become resistant and exhibit further tumor progression despite their initial response (4). Thus, it remains important to search for novel agents for new combination treatments to enhance efficiency, reduce toxicities and avoid the progression of colorectal tumors.

As a key factor contributing to tumor survival and progression during colorectal cancer therapy (5,6), NF-κB can be activated by CPT-11, it is already upregulated in most colorectal cancers during early treatment (7) and could be a potential chemo-resistance mechanism in malignant cells (8,9) that will reduced chemosensitivity. NF-κB activation is regulated by proteasomes, which are an attractive target for cancer therapy, as the proteasome plays a central role in the regulation of proteins that modulate cell cycle progression, cell survival, migration and direct induction of apoptosis (10,11). There are various kinds of proteasome inhibitors which can be classified by different structures and reaction mechanisms (12), such as bortezomib (PS-341), carfilzomib (CFZ), NPI0052, MLN-9807 and CEP-18770, it is encouraging that all these proteasome inhibitors block activation of NF-κB that is one of the most important mechanisms for killing transformed tumor cells and is the foundation of rational combination therapy (13). In the study by Tamatani *et al*, PS-341 enhanced radiosensitivity through inhibiting radiation-induced NF-κB activity and suppressed oral tumor growth (14). Moreover, PS-341 and NPI0052 can enhance chemosensitivity and the tumoricidal response to CPT-11 in colorectal cancer by blocking chemotherapy-induced NF-κB activation and expression of genes involved in cancer cell survival (15–18). In addition, proteasome inhibition also augments the cancer cell response to chemotherapy and radiation by modulating other NF-κB related proteasome-dependent regulatory proteins involved in treatment resistance such as Bcl2, p53, the caspases and stress response molecules like SAPK/JNK, as well as accumulation of misfolded proteins and anti-angiogenic effects (10,13).

Carfilzomib (CFZ) is an epoxyketone proteasome inhibitor that irreversibly inhibits the 26S proteasome, and has high specificity for inhibiting chymotrypsin-like activity (19–21). In preclinical studies, CFZ has shown single-agent activity against hematopoietic malignancies and some solid tumors, such as head and neck cancer, through inhibiting NF-κB activation by preventing ubiquitination and proteasome degradation of IκBα as well as through other NF-κB related biological mechanisms (22–25). This novel second-generation proteasome inhibitor was approved by the FDA in July of 2012 and is in clinical use for the treatment of relapsed and refractory multiple myeloma (MM), it can be used safely and effectively in place of PS-341 combination therapy, especially in patients with PS-341 resistant MM (26), and CFZ has no effect on normal skin or normal umbilical vein cells and is less toxic to normal peripheral blood mononuclear cells from healthy individuals than to tumor cells (19,25). CFZ is drawing increasing attention, but little is known about its activity against CRC and it has not yet been fully evaluated. In addition, the combination effect of CFZ and CPT-11 in colorectal cancer treatment is not known.

These results led us to hypothesize that CFZ, which is more efficacious and less toxic to patient with haematological malignancies than earlier proteasome inhibitors, could block CPT-11 induced NF-κB activation and mediate apoptosis pathways to improve the effectiveness of CPT-11, leading to a dramatic augmentation of chemosensitivity. In the present study, we examine the therapeutic ability of CFZ combined with CPT-11 *in vitro* and *in vivo* by evaluating the effect on CRC tumor growth, cell proliferation, cell cycle progression, apoptosis, migration and invasion, as well as on NF-κB regulated pathways. Our results indicate that CFZ and CPT-11 interact synergistically in SW620 cells *in vitro* and *in vivo* through a process that involves NF-κB inhibition that is related to the apoptotic response.

## Materials and methods

### Cell lines and culture

Human colorectal cancer cell lines, SW620 and HCT8 were obtained from the Cell Bank of the Type Culture Collection of the Chinese Academy of Sciences (Shanghai, China). SW620 was cultured in L-15 medium and HCT8 was maintained in RPMI-1640 medium, both nutrient media (Gibco, USA) were supplemented with 10% fetal bovine serum (Gibco). Cells were grown at 37°C with saturating humidity.

### Drugs and antibodies

Carfilzomib was purchased from Biorbyt Ltd. (Cambridge, UK) and CPT-11 from Tocris Bioscience (Bristol, UK). Both agents were maintained in dimethyl sulfoxide for *in vitro* studies, CFZ was in 10% captisol (sulfobutylether-β-cyclodextrin) in 10 mmol/l citrate buffer pH 3.5 and CPT-11 was dissolved in sterile water for *in vivo* studies. Antibodies against TRAF6, BCL10, IKKs, phospho-IκBα/IκBα, NF-κB (p65/p52/p50), phospho-NF-κB p65, MEK, phospho-MEK (Ser217/221), ERK1/2, phospho-ERK1/2 (p44/42 MAP kinase, Thr202/Tyr204), SAPK/JNK, phospho-SAPK/JNK (Thr183/Tyr185), PI3K, phospho-PI3 kinase p85 (Tyr458)/p55 (Tyr199), AKT, phospho-AKT (Ser473), PCNA, survivin, Stat5, phospho-Stat5 (Tyr694), Stat3, phospho-Stat3 (Tyr705), p53 and β-tubulin were from Cell Signaling Technology Inc. (Beverly, MA). Antibodies against β-catenin, cdc25c, cyclin D1 (M20), cyclin B1 (H20), cyclin A (C-19), Cdk1 (C-19), phospho-Cdk1 (Thr14/Thr15), Cdk2 (M2), phospho-Cdk2 (Thr160), p21 (WAF1/CIP), PARP, p38, phospho-p38 (Thr180/Tyr182), ATF3, MMP1, MMP2, MMP9, TIMP1, Egr1 and β-actin were from Santa Cruz Biotechnology (Santa Cruz, CA, USA). Anti-MKP-1 was from Merck Millipore (Bedford, MA, USA).

### WST-1 test for cell proliferation assay

The cytotoxicity of CFZ and CPT-11 on SW620 and HCT8 cells was tested using the WST-1 cell proliferation assay (27). Cells (1×l0^4^ cells per well) were plated overnight in 96-well microplates (Costar, Corning, NY, USA) with 100 μl culture medium and then treated with CFZ or CPT-11 at various concentrations. After various periods of incubation, 10 μl of WST-1 reagent (Roche, Germany) was added to each well and incubated with cells at 37°C for 4 h, and plates were read on a microplate reader (Bio-Rad, model 550) at 450 nm with a reference wavelength at 630 nm after being shaken thoroughly, as described previously (28).

### Clonogenic assay

A clonogenic assay was performed with SW620 cells, 500 cells per well were plated in 6-well plates in L-15 medium supplemented with 10% fetal bovine serum. The cells were treated with CFZ and CPT-11. The number of colonies (>50 cells) was counted after 14 days incubation at 37°C.

### Cell cycle analysis and apoptosis assay by flow cytometry (FACS)

The CycleTESTy Plus DNA reagent kit from Becton-Dickinson Immunocytometry Systems was used to test cell cycle distribution. According to the manufacturer’s instructions, the cells were treated with trypsin buffer, trypsin inhibitor, RNase buffer and propidium iodide (PI) stain solution. The cells were evaluated on a FACSCalibur (BD Biosciences) and results analysed with Cell Quest and ModiFit software; analysis of phosphatidyl serine (PS) was performed as described in the Annexin V apoptosis detection kit (BD Biosciences). Briefly, SW620 cells treated with different concentrations of drugs were harvested, labelled with Annexin V and PI, and analyzed with a FACSCalibur flow cytometer. For caspase 3 expression, SW620 cells were treated with permeabilizing solution and incubated with FITC anti-caspase 3 antibody. CD95 expression was detected by direct labelling with anti-CD95 antibody.

### Terminal deoxynucleotidyltransferase-mediated TMR red-dUTP nick end labelling (TUNEL) experiment

TUNEL assays were performed according to the manufacturer’s protocol with the In Situ Cell Death Detection Kit (TMR red; Roche, Germany). For the *in vitro* cell assay, after fixing with 4% paraformaldehyde/PBS, cells were incubated with permeabilisation solution (freshly prepared; 0.1% Triton X-100 in 0.1% sodium citrate) on ice (2–8°C). Cells were washed twice with PBS, and resuspended in the TUNEL reaction mixture (terminal deoxynucleotidyl transferase enzyme with digoxigenin-nucleotide), and incubated for 1 h at 37°C. The incorporation of nucleotides into 3′-DNA through cleavage of DNA during apoptosis was detected by a TMR red staining system. The cells were analyzed by fluorescence microscopy; for paraffin-embedded tissue, after dewaxing and rehydration, tissue slices were incubated with permeabilization solution and rinsed twice with PBS, and then processed using the protocol described for cells according to the manufacturer’s instructions.

### NF-κB activity assay - electrophoretic mobility shift assay (EMSA)

Nuclear proteins from *in vitro* treated cells were extracted with the Norvagen NucBuster protein extraction kit (EMD Biosciences, affiliate of Merck KgaA, Germany). For release of nuclei, cell pellets were suspended in NucBuster extraction reagent I, 50 μl of packed cell volume suspended in 150 μl of reagent I on ice, then centrifuged to remove the cytoplasmic fraction by washing with ice-cold 1X PBS (137 mM NaCl, 43 mM Na_2_HPO_4_, 27 mM KCl, 15 mM KH_2_PO_4_, pH 7.3). The nuclei were harvested and suspended in 50 μl of NucBuster extraction reagent II on ice and centrifuged at 16,000 × g for 5 min at 4°C in order to separate nuclear extracts. Nuclear proteins from *in vivo* treatment of colorectal tumor tissues were extracted using the protocols from the NE-PER Nuclear and Cytoplasmic Extraction kit (Pierce Thermo Scientific, USA). A total of 7 μg of protein extract was subjected to electrophoretic mobility shift assay for NF-κB/DNA binding using a DIG label as per the protocol in the DIG Gel Shift kit (Roche, Germany). Oct-1 or Oct-2A was used as a loading control in EMSA.

### Western blot analysis

Cells were lysed in modified RIPA buffer (Roche) for 30 min at 4°C, then the cell lysates were cleared of debris by centrifugation and they were mixed in an equal volume of sample buffer (0.2% bromophenol blue, 20% glycerol, 125 mM Tris-HCl, 640 mM βME, 4% SDS), then boiled for 10 min. The Bradford assay was used to determine the protein concentration, and bovine serum albumin (BSA) (Sigma) was used as the standard. Protein samples, 25 μg, were separated on 8 or 10% SDS-PAGE and then the proteins were transferred onto nitrocellulose membranes (Amersham, UK). Membranes were blocked at room temperature for 1 h and incubated overnight with the appropriate antibody at 4°C, followed by three washings with Tris-buffered saline containing 0.1% Tween-20 (TBS-T). Membranes were incubated for 2 h with secondary antibody (anti-rabbit or anti-mouse; diluted 1:10,000) at room temperature. Tubulin or actin was assayed as protein loading controls. Chemiluminescence reagent (ECL Western Blotting Detection System, Amersham, UK) was used to detect the expression of proteins.

### Wound-healing assay

SW620 cells were seeded into 12-well plates, a line wound was scraped in the confluent adherent cells using a pipette tip in each well and the plate washed with PBS. The cells were then cultured with serum-free medium. After treatment with the indicated drugs, the scraped line was observed and photographed in three randomly selected views in each treatment well. The reduction of the scraped area was considered as wound-healing induced by cell migration.

### Transwell cell migration/Matrigel penetration assays

Transwells with 8 μm pore polycarbonate membranes (Costar, Corning) were left uncoated or were coated with matrix before use in simple migration assays and invasion assays, respectively. Matrigel (BD Biosciences) were added to the Transwell inserts and polymerised at 37°C, then SW620 cells in 1% serum-containing medium were seeded into the upper wells of the transwell, and 20% serum-containing medium was added to the outer wells. In order to verify that cells did not proliferate during the assay, cells were also seeded into tissue culture dishes under the same condition. Following treatment with the indicated drugs for 48 h, the migrating or invading cells were fixed and stained with crystal violet, then photographed and counted in three randomly selected views using a microscope.

### Animal studies

Five to six weeks old female athymic BALB/c nude mice were purchased from the Vital River Laboratory Animal Technology Co., Ltd. (Beijing, China). SW620 cells, 10×10^6^, were inoculated into the backs of mice and treatment began when the diameter of tumor reached 7 mm. Mice were randomly grouped into four sets with five mice in each set: i) control group, treated with vehicle alone, 10% Captisol in 10 mmol/l citrate buffer, (i.v. twice weekly for three weeks on days 1 and 2); ii) CFZ group, 2.0 mg/kg (i.v. twice weekly for three weeks on days 1 and 2); iii) CPT-11 group, 33 mg/kg (i.p. once weekly for four weeks on day 1); and iv) combined group, CFZ in combination with CPT-11. Tumor size was measured twice per week and the volume calculated as follows: V=(a × b^2^)/2, where ‘a’ and ‘b’ are the largest and smallest diameters, respectively. After the final treatment, mice were sacrificed and tumor were excised and weighed. All experimental procedures and protocols were approved by the Animal Ethics Committee of Fuzhou General Hospital.

### Statistical analysis

The comparison of differences between control and treated groups was performed using a 2-tailed Student’s t-test carried out with SPSS 11.0 statistical software (SPSS, Inc., Chicago, IL, USA). P<0.05 was considered significant. Synergistic and antagonistic interactions were defined by median dose effect analysis with a commercially available software (CompuSyn) (29). Combination index (CI) scores of <0.7, 0.7–0.85, 0.85–0.90, 0.90–1.10 and >1.10 indicate synergism, moderate synergism, slight synergism, additive effect and antagonism, respectively.

## Results

### Carfilzomib interacts synergistically with CPT-11 on SW620 cells

**Figure 1:**
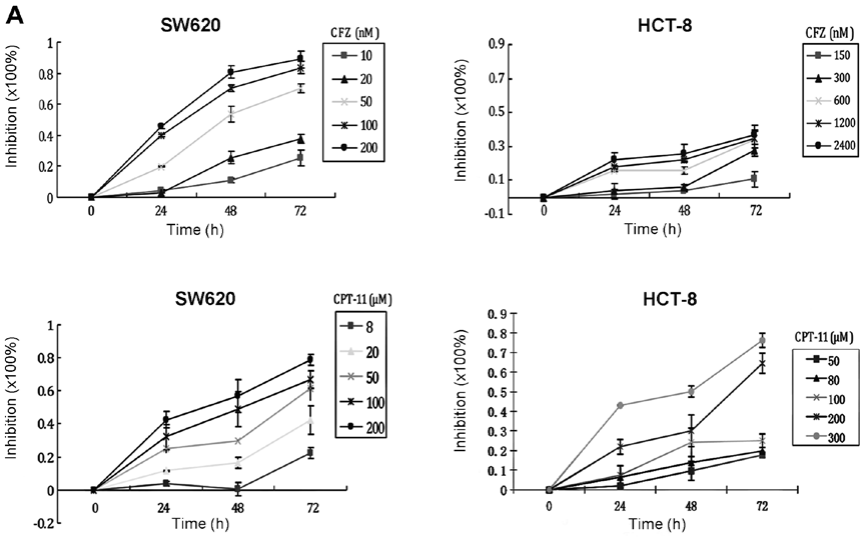

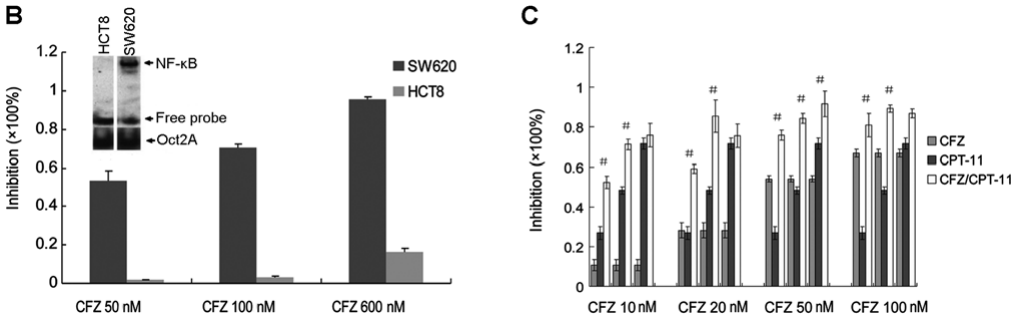

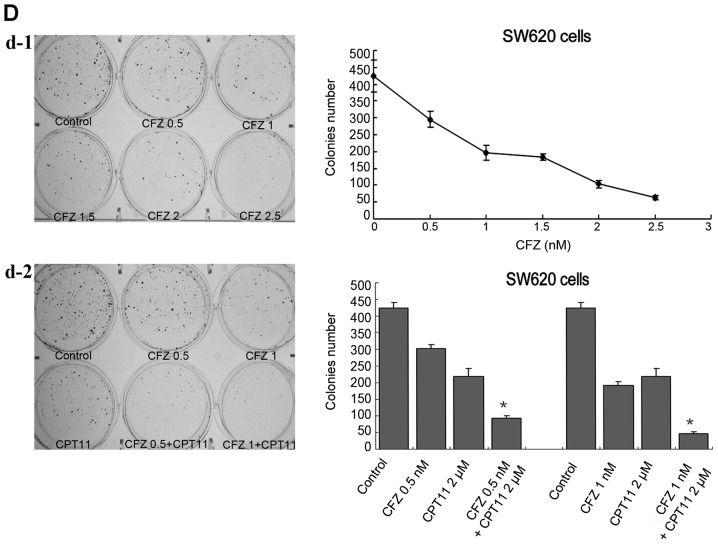
CFZ interacts synergistically with CPT-11 on CRC cells. (A) Inhibitory effect of CFZ on human colorectal cancer cell proliferation. SW620 cells were treated with 10, 20, 50, 100 and 200 nM of CFZ and 8, 20, 50, 100 and 200 μM of CPT-11 for 24, 48 and 72 h. HCT8 cells were treated with 150, 300, 600, 1,200 and 2,400 nM of CFZ and 50, 80, 100, 200 and 300 μM of CPT-11 for 24, 48 and 72 h. The WST-1 assay determined cell proliferation. (B) Using the same concentration of CFZ (50, 100 and 600 nM) on SW620 cells and HCT8 cells, we find that SW620 cells were more sensitive to CFZ than HCT8. NF-κB was detected by EMSA. (C) CFZ and CPT-11 exhibit synergistic cytotoxicity on SW620 cells. SW620 cells were treated with CFZ and CPT-11 at the indicated concentrations for 48 h. Each CFZ concentration was combined with 50, 100 and 200 μM of CPT-11 as showed in Table 1. Cell viability was measured using the WST-1 assay. Effects of CFZ and CPT-11 on the colony formation of SW620 cells. The colonies (>50 cells) were scored after 14 days. (d-1) SW620 cells were treated with 0.5, 1, 1.5, 2 and 2.5 nM of CFZ, colony number decreased as the concentration of CFZ increased. (d-2) SW620 cells were treated with 0.5 or 1 nM of CFZ and with 2 μM CPT-11. The number of colonies significantly decreased compared to CFZ or CPT-11 treatment alone. All the above data shown represent the mean ± SEM (n=3).

To verify the cytotoxicity of CFZ on CRC cells, SW620 cells, in which NF-κB is constitutively activated, and HCT8 cells, in which NF-κB is not constitutively activated, were exposed to increasing concentrations for various times and assessed by the WST-1 assay. The results demonstrated that CFZ reduced cell viability in a concentration-dependent and time-dependent manner (Fig. 1A). The IC_50_ of CFZ for inhibiting SW620 proliferation at 24, 48 and 72 h were 201.06±4.38, 50.26±1.38 and 27.06±0.31 nM, respectively. The IC_50_ of CFZ inhibition of HCT8 cells was more than 2,500 nM at 24, 48 and 72 h. At concentrations of 50, 100 and 600 nM, the inhibition of SW620 cell proliferation by CFZ was significantly higher than inhibition of HCT8 cells (Fig. 1B).

To determine whether treatment with CFZ would enhance the anticancer effects of CPT-11 chemotherapy, human SW620 cells were exposed to various concentrations of CFZ, CPT-11 or combinations of the two drugs. As shown in Table I, when we give a concentration of 10 nM CFZ in combination with 50 μM CPT-11, inhibition reaches 52.29±3.17%, which is equal to or better than the effect of 100 μM CPT-11 alone (48.19±1.84%), and much better than single-drug effect of 50 μM CPT-11 (26.87±3.28%). Similarly, if we add 20 nM CFZ to CPT-11 treatment of SW620 cells, we can reduce the CPT-11 dosage to 100 μM and achieve a better inhibition rate, 85.39±8.11%, than a 200 μM CPT-11 treatment alone, 71.63±2.91%. In order to achieve equivalent inhibition to 200 μM CPT-11 (71.63±2.91%), while reducing the dose of drugs in a combination regimen as much as possible, we found that 50 nM CFZ combined with 50 μM CPT-11 will produce a 75.94±2.57% inhibition rate. When we increased the concentration of CPT-11 to 100 μM with 50 nM CFZ, single drug concentrations that produced about a 50% inhibition rate, the inhibition effects of the combination regimen reached almost the best, and higher doses produced a plateau of inhibition. When CFZ was used as a single-agent treating SW620 cells at 10, 20, 50 and 100 nM for 48 h, we recorded 10.62±2.78, 28.16±3.75, 54.09±1.55 and 66.98±1.99% growth inhibition, respectively. When CPT-11 was combined with CFZ, the growth inhibition was improved significantly, compared with single agent CPT-11; a synergistic action was observed with all dose of CFZ added to 50 and 100 μM of CPT-11 (CI<0.9). An additive effect was seen with the 200 μM dose of CPT-11 with all doses of CFZ except 50 nM (0.90<CI<1.10; Fig. 1C). These data show that the inhibition of proliferation of SW620 cells by CPT-11 is greater when combined with CFZ.

Similar effects were observed in the clonogenic assay with SW620 cells (Fig. 1D). Co-administration of 0.5–1.0 nM of CFZ with 2 μM of CPT-11 sharply decreased colony formation of SW620 cells. These results demonstrate an inhibitory effect of CFZ on proliferation and colony formation of SW620 cells, and that the cell growth inhibition of CPT-11 was enhanced by CFZ.

### Inhibition of NF-κB activation plays a key role in CFZ enhanced chemosensitivity of SW620 cells to CPT-11

SW620 cells, in which NF-κB is activated, were used to assess the functional role of NF-κB activity during CFZ/CPT-11 inhibition of CRC cell growth. As shown in Fig. 1B the SW620 cells were more sensitive to CFZ than were HCT8 cells, in which NF-κB was shown by EMSA to be not activated. This suggests that NF-κB may play a significant role in CFZ induced lethality.

**Figure 2:**
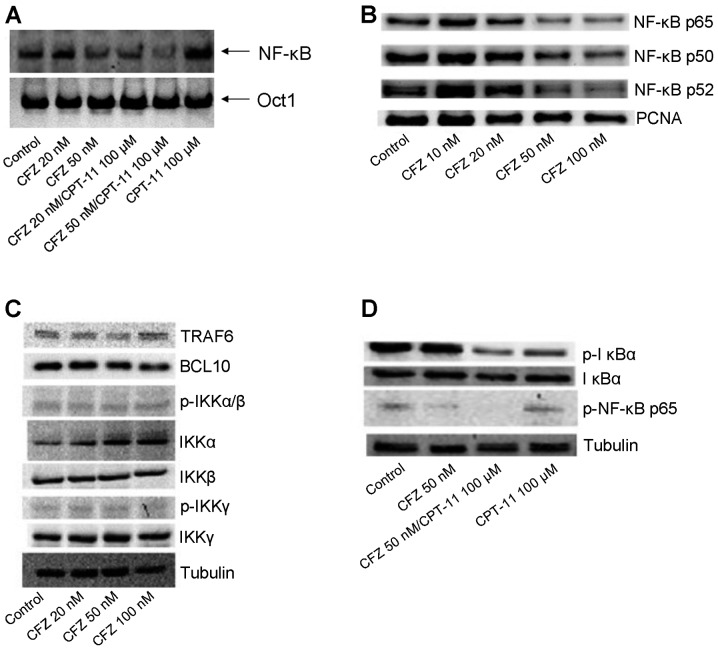
CFZ alone or combined with CPT-11 inhibits NF-κB activation in SW620 cells. (A) EMSA was used to evaluate the effect of CFZ (20 and 50 nM) ± 100 μM CPT-11 treatment in SW620 cells. Cells were harvested and the nuclear proteins of the cells were extracted and assayed for nuclear translocation of NF-κB at 48 h after of chemotherapy treatment. An Oct1 probe was used as a control for EMSA. (B) The nuclear proteins from SW620 cells were extracted after treatment with 10, 20, 50 and 100 nM of CFZ for 48 h. NF-κB activation was analyzed by western blot analysis. PCNA was used as the internal control for nuclear protein. With increasing concentrations of CFZ, the nuclear protein expression of NF-κB p65, p50 and p52 were gradually reduced. (C) SW620 cells were incubated with 20, 50 and 100 nM of CFZ for 48 h, whole cell lysates were collected and subjected to western blot analysis. IKKα was moderately upregulated but TRAF6, BCL10, p-IKKα/β, IKKβ, p-IKKγ and IKKγ were not changed. (D) After 48 h of chemotherapy treatment, we probed total protein preparations by immunoblot analysis using anti-phospho-IκB, anti-IκB and anti-phospho-NF-κB p65. Tubulin was used as an internal control. CPT-11 (100 μM) combined treatment with CFZ (50 nM) in SW620 cells sharply decrease p-NF-κB p65 activation, and also decrease p-IκB activation but had no effect on the total IκB protein.

An EMSA and western blot analysis assay showed that CPT-11 may induce slightly the NF-κB activation in SW620 cells (Fig. 2A and 2D). Treatment with CFZ alone or combined with CPT-11 resulted in a decrease of NF-κB by blocking the degradation of IκBα (Fig. 2A, B and D), although there is significantly less of the phosphorylated form of IκBα and total IKKα was slightly upregulated, IκBα protein was not completely degraded, and may still bind to NF-κB. There was no effect on IκB upstream genes, such as TRAF6, BCL10 or other IκB kinase complexes (IKKs) (Fig. 2C). Thus, we conclude that CFZ co-administration reduced NF-κB DNA binding in CPT-11-treated SW620 cells.

### CFZ combined with CPT-11 attenuates the MEK/ERK pathway, accompanied by PI3K/AKT pathway dephosphorylation and survivin downregulation in SW620 cells

**Figure 3:**
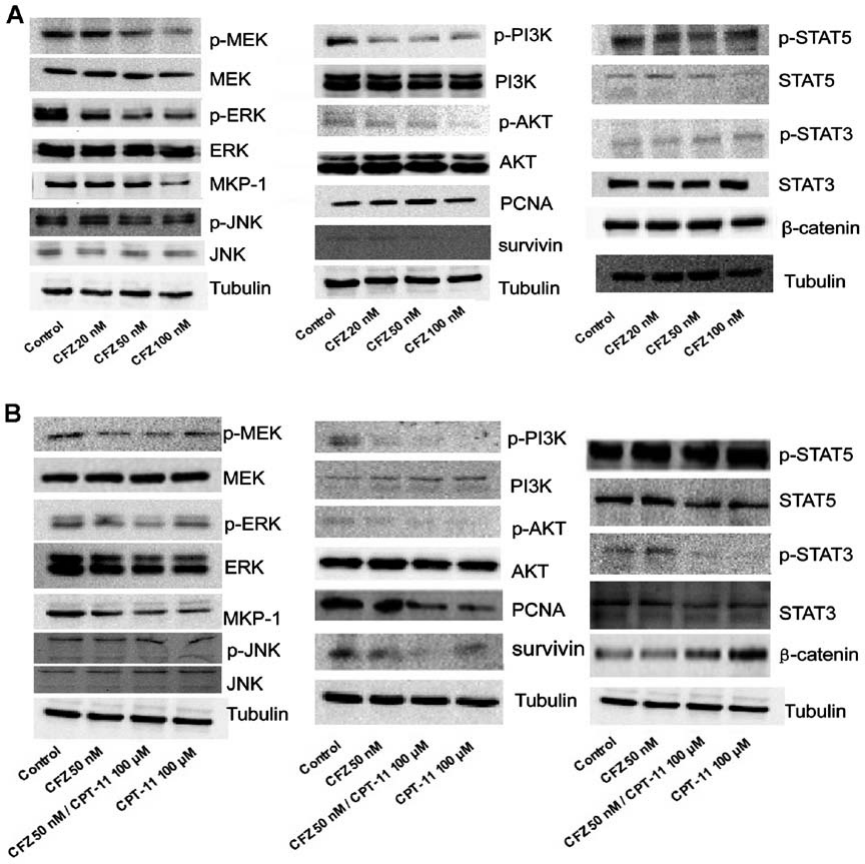
CFZ alone or combined with CPT-11 blocks the phosphorylation of MEK/ERK and PI3K/AKT pathway, as well as expression of MKP-1 and survivin in SW620 cells. (A) SW620 cells were treated with increasing doses of CFZ (20, 50 and 100 nM) for 48 h. (B) SW620 cells were treated with 100 μM CPT-11 combined with 50 nM CFZ for 48 h. Protein expression was monitored with the indicated antibodies by western blot analysis as described in Materials and methods. Tubulin was used as the loading control.

To determine whether the combination chemotherapy or CFZ alone induced growth inhibition of SW620 cells was associated with multiple mechanisms, such as blocking other survival pathways, CFZ at 20, 50 and 100 nM, and 100 μM of CPT-11 combined with 50 nM of CFZ was used to treat SW620 cells for 48 h. In the stress-related MAP kinase signalling modules, 100 μM of CPT-11 alone had little effect, while exposure of cells for 48 h to CFZ, with or without CPT-11, reduced phospho-MEK and phospho-ERK expression as well as that of MKP-1. Levels of total MEK and total ERK and JNK pathway related proteins were unchanged. Perturbations were also observed in the cytoprotective-related PI3K/AKT pathway. Administration of CFZ alone to SW620 cells decreased phospho-PI3K and phospho-AKT, and decreased the expression of survivin. Combined treatment with CPT-11 also resulted in a marked decrease in phosphorylation of PI3K and AKT, as well as in expression of downstream survivin. Total PI3K and AKT levels were unchanged under various concentration of CFZ treatment and there was little effect from administration of individual agents. CFZ alone exerted a minimal effect and CPT-11 contributed more to the combined effect in lowering the expression of STAT5, phospho-STAT3, STAT3, PCNA and enhancing the expression of β-catenin, and treatments failed to produce changes in other STAT-related pathway molecules (Fig. 3A and B).

Together, these findings indicate that administration of CFZ and CPT-11 or CFZ alone leads to MEK/ERK pathway inactivation and MKP-1 downregulation, accompanied by PI3K/AKT pathway dephosphorylation and survivin inhibition.

### Combined CFZ with CPT-11 or CFZ alone induces G2/M arrest

**Figure 4:**
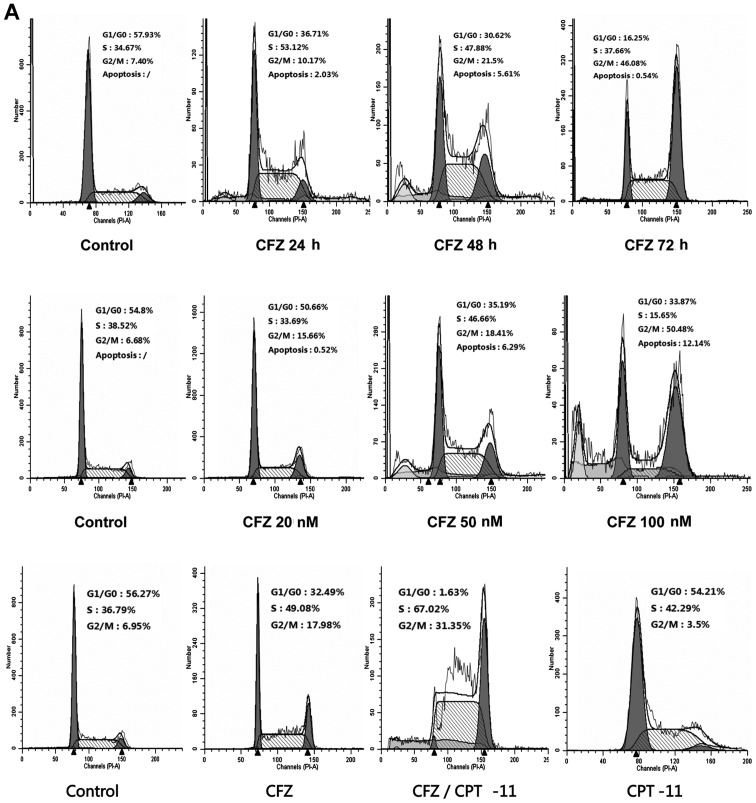

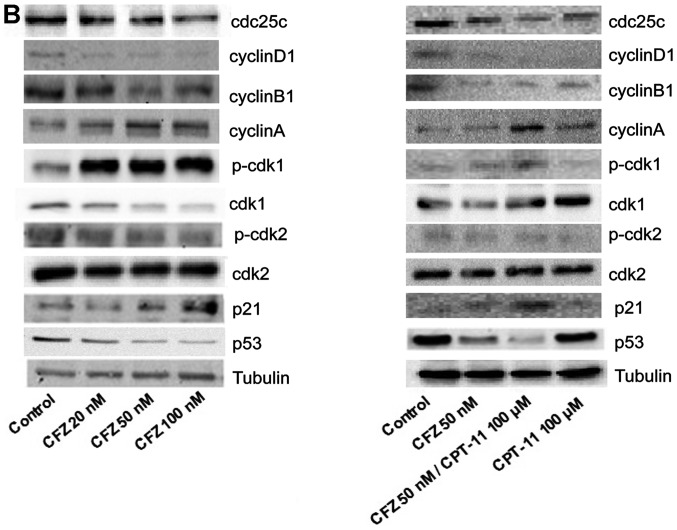
Combined CFZ with CPT-11 or CFZ alone induces G2/M arrest. (A) Cell cycle analyzed by FCM of SW620 cells. Cells were treated with 50 nM of CFZ for 24, 48 and 72 h, and varying doses of CFZ (20, 50 and 100 nM) for 48 h. In the combined regimen, cells were exposed to CFZ (50 nM) and CPT-11 (100 μM) for 48 h. (B) Expression of cell cycle related proteins in SW620 cells. Cells were treated with various doses of CFZ, or with CFZ ± CPT-11 for 48 h, then the total protein were extracted for western blot analysis. Both CFZ alone or in combination with CPT-11 decreased the expression of cdc25c, cyclin D1, cyclin B1, cdk1 and mutant p53, and increased the expression of cyclin A, phospho-cdk1^Thr14/Thr15^ and p21^WAF1/CIP^, but had no notable effect on the level of phospho-cdk2 or cdk2.

To further elucidate the mechanism of growth inhibition and whether the cell cycle changed under CFZ and combined regimens, SW620 cells were treated with 50 nM CFZ for 24, 48 and 72 h, and exposed to various concentrations of CFZ (20, 50 and 100 nM) for 48 h. Both resulted in a change in the cell cycle profile. CFZ alone induced G2/M arrest and CFZ combined with CPT-11 also resulted in a pronounced accumulation of cells in G2/M (Fig. 4A). Before treatment 7.4% of cells were in G2/M. After 72-h treatment with 50 nM CFZ, 46.08% of cells were in G2/M. The percentage of cells in G2/M phase increased in a dose-dependent manner from 6.68 to 50.48% after treatment with CFZ for 48 h. The percentage of cells in G0/G1 decreased from 54.8% in untreated cells to 33.87% for cells treated with 100 nM CFZ. CFZ treatment led to significant concentration-dependent and time-dependent G2/M arrest without a change in S phase. In the combined regimen there was also a shift toward G2/M arrest compared with the untreated controls, 6.95 vs. 31.35%. These results demonstrate that the inhibition of SW620 cell proliferation proceeds via G2/M-phase arrest. To determine what effect combined exposure of SW620 cells to CFZ and CPT-11 would have on various cell cycle regulatory proteins, cells were exposed for 48 h to various concentrations of CFZ and to 50 nM CFZ combined with 100 μM CPT-11, after which expression of cyclins, CDKs, CKIs and cdc25c were monitored by western blot analysis. Both CFZ treatment alone or combined with CPT-11 resulted in reduction in the levels of cdc25c, cyclin D1 and cyclin B1, as well as cdk1 and mutant p53 (Fig. 4B). There was an increase in the levels of cyclin A, p-cdk1^Thr14/Thr15^ and p21^WAF1/CIP^ (Fig. 4B), which may explain this shift toward G2/M arrest.

### CFZ increases apoptosis and necrosis induced by CPT-11 in SW620 cells by inducing caspase 3 and CD95 upregulation, as well as p-p38 and ATF3 activation

**Figure 5:**
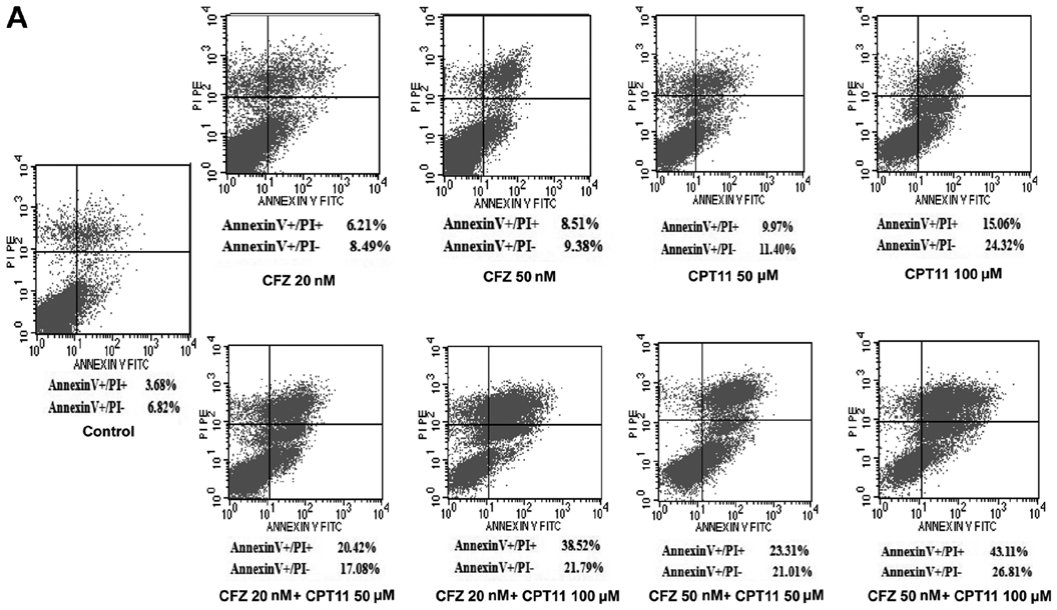

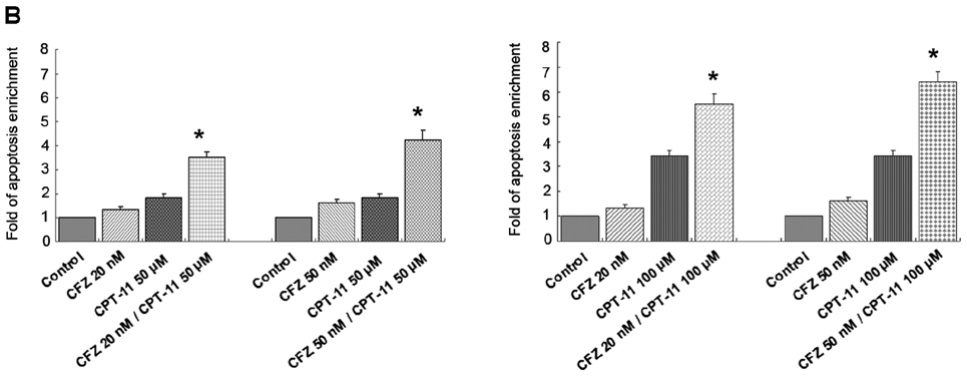

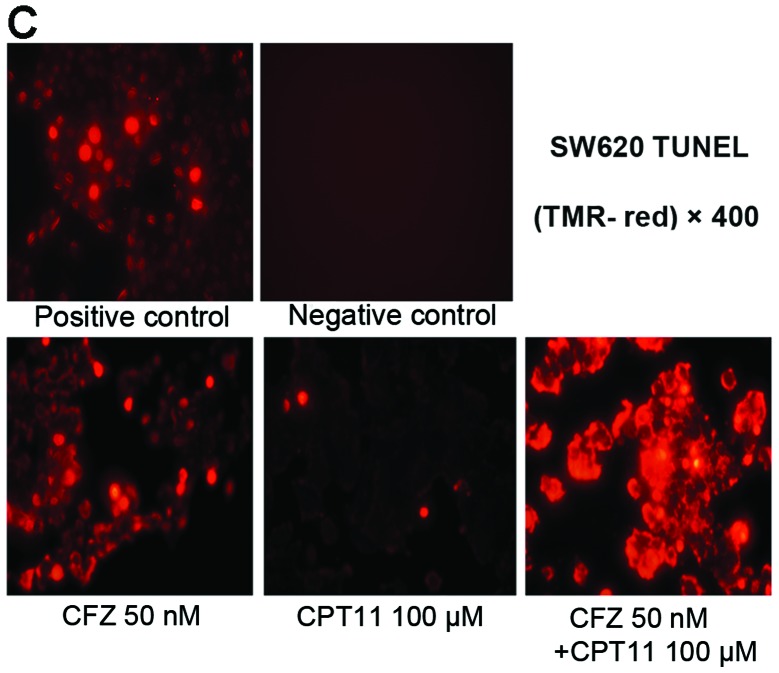

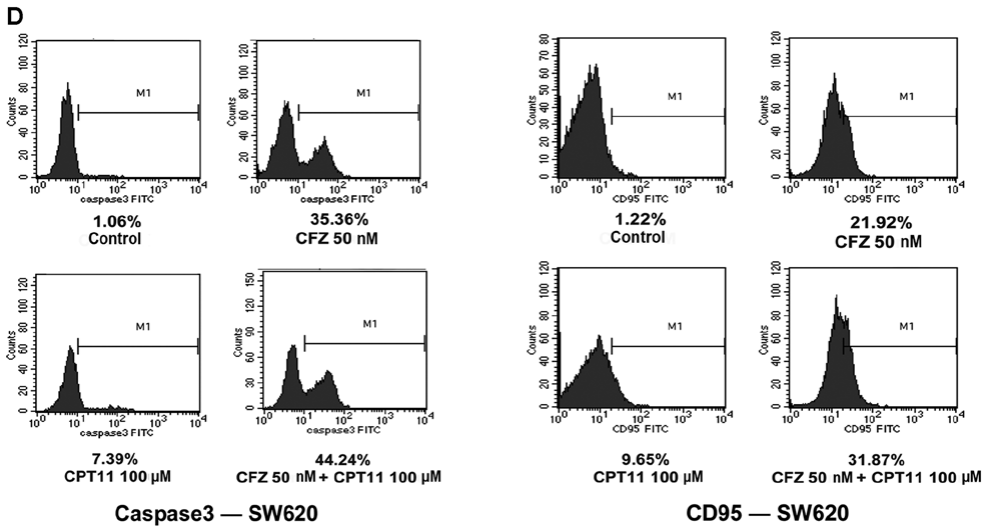

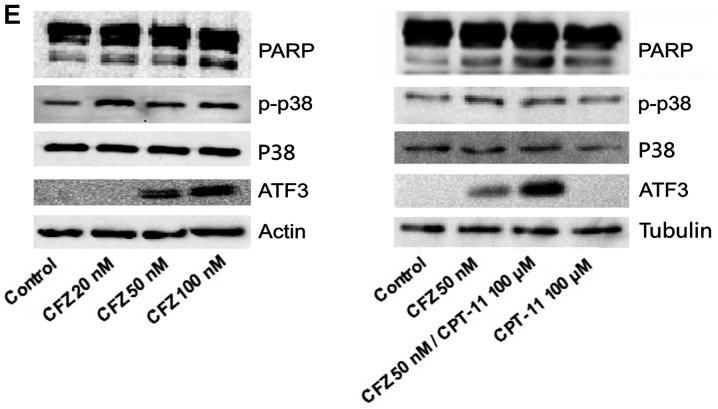
CFZ ± CPT-11 induces apoptosis by caspase 3 and CD95 upregulation, as well as p-p38 and ATF3 activation in SW620 cells. (A) The effect of CFZ ± CPT-11 on the expression of Annexin V/PI in SW620 cells. SW620 cells were treated with 20 and 50 nM CFZ alone or in combination with 50 and 100 μM CPT-11 for 48 h. The results show the percentages of apoptosis (Annexin V^+^/PI^−^) and necrosis (Annexin V^+^/PI^+^). (B) SW620 cells were treated with the indicated concentration of CFZ ± CPT-11 for 48 h, and cell death was determined by Annexin V/PI (part of the data from Fig. 5A; bars, SD, n=3). ^*^P<0.05 significantly greater than values obtained for cells treated with CPT-11 alone. (C) Representative photomicrographs demonstrating TUNEL labelling of SW620 cells after treatment with CFZ or CPT-11 alone, and combination CFZ and CPT-11. Cells were harvested 48 h after final treatment. TUNEL positive cells (fluorescent red) were detected using a fluorescence microscope. Positive controls were treated with DNaseI for 10 min at 15–25°C. Negative controls were treated without terminal deoxynucleotidyl transferase. (D) The effect of CFZ ± CPT-11 on the expression of caspase 3 and CD95. SW620 cells were treated with 50 nM CFZ alone or in combination with 100 μM CPT-11 for 48 h. The results showed the percentages of caspase 3 and CD95 expression. (E) SW620 cells were exposed to increasing concentrations of CFZ (20, 50 and 100 nM) and combined agents (50 nM CFZ and 100 μM CPT-11) for 48 h. Total protein expression was monitored by western blot analysis with the indicated antibodies.

To investigate whether CFZ was able to reduce cell viability by apoptosis, Annexin V/PI analysis and TUNEL were performed to determine whether apoptosis could be responsible for the synergistic effect of the combined treatment of SW620 cells. Notably, the Annexin V/PI test showed that administration of low-middle doses of CFZ (20 and 50 nM) and CPT-11 (50 and 100 μM) resulted in pronounced apoptosis in SW620 cells (Fig. 5A). The average percent of apoptotic cells increased significantly after exposure to single agents compared with the control (P<0.05). The combination treatment produced the greatest increase in both necrotic and apoptotic SW620 cells. Apoptosis induced by combination treatment was 1.62–2.30-fold higher in SW620 cells, compared with CPT-11 alone (Fig. 5B). The TUNEL assay of SW620 cells revealed that, whereas CPT-11 had little effect and CFZ alone modestly increased TUNEL labelling, combined treatment resulted in a pronounced increase in TUNEL labelling (Fig. 5C). Western blot analysis revealed that combined treatment as well as CFZ treatment also resulted in a striking increase in PARP cleavage, which is indicative of apoptosis (Fig. 5E).

To elucidate the molecular mechanism by which CFZ or the combination regimen induces apoptosis in SW620 cells, we assessed caspase 3 activity and CD95 expression. CFZ combined with CPT-11 resulted in a greater increase in the caspase 3 activity and CD95 expression (Fig. 5D), accompanied by marked increases in p-p38 and ATF3 activation, without changes in total p38 (Fig. 5E). This indicates that the p-p38/ATF3 pathway, as well as caspase 3 activation and CD95 upregulation, may play a significant functional role in CFZ/CPT-11 lethality in SW620 cells.

### The invasion and migration inhibitory effects of CFZ or CFZ/CPT-11 in vitro

**Figure 6:**
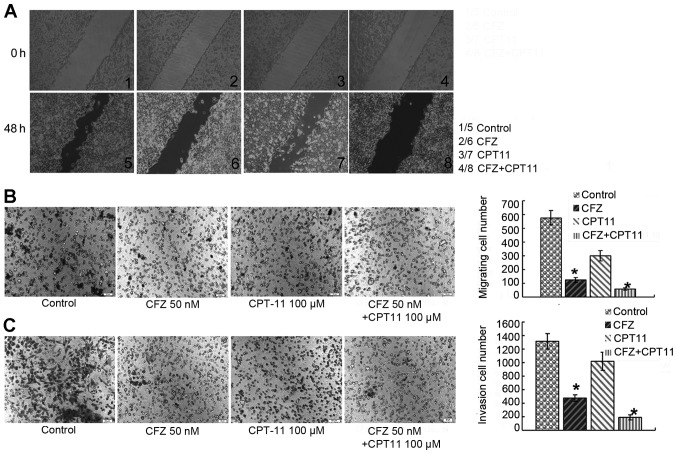

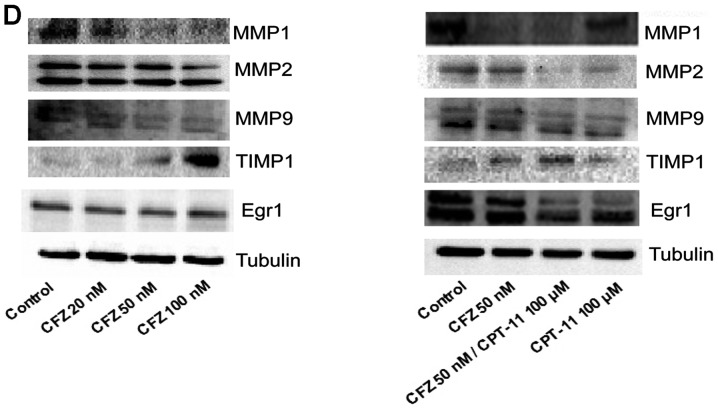
CFZ ± CPT-11 decreases SW620 cell invasion and migration via reduced MMPs and increased TIMPs. (A) Effect of CFZ±CPT-11 on SW620 migration was performed by a wound healing assay. After treatment with the indicated concentrations of CFZ and CPT-11 for 48 h, the migration of SW620 cells was observed using phase contrast microscopy. The images were obtained at ×200 magnification. (B) A Transwell membrane migration assay with SW620 cells. After 48 h, cells were fixed, stained and counted. CFZ alone or combined with CPT-11 significantly decreased cell migration. Representative photographs show cells. ^*^P<0.001 significantly less than control. (C) A Transwell Matrigel invasion assay was done with SW620 cells. After 48 h, the cells were fixed, stained and counted. CFZ alone or combined with CPT-11 led to significant decreases in cell invasion. ^*^P<0.001 significantly less than the control. (D) Changes in MMPs and TIMPs with CFZ and CPT-11 treatment. SW620 cells were treated with CFZ, CPT-11 or CFZ plus CPT-11. Cell lysates were subjected to western blotting for analysis of MMPs and TIMPs levels.

To assess the inhibition of invasion and migration of CFZ ± CPT-11 *in vitro*, its effects on the motility of SW620 cells were evaluated in the wound-healing assay. After 48 h, untreated SW620 cells migrated into the wounded area on the plate, whereas the migration of cells under 50 nM CFZ treatment was inhibited. Combined treatment with 50 nM CFZ and 100 μM CPT-11 resulted in a significant decrease in migration of SW620 cells compared to CFZ or CPT-11 alone (Fig. 6A). The effect of CFZ on SW620 cell migration across the transwell membrane and invasion through a Matrigel was also assayed. The combination of 50 nM CFZ and 100 μM CPT-11 led to a decrease in cell migration (Fig. 6B) and cell invasion (Fig. 6C), which was significantly greater than the decrease with either CFZ or CPT-11 alone.

To further investigate the underlying mechanism of CFZ invasion and migration inhibitory activity, the effect of CFZ with and without CPT-11 on MMPs, TIMPs and Egr1 in SW620 cells was investigated. The results of the western blot analysis showed that both CFZ alone and combination treatment reduced MMP1 and MMP9 protein expression and increased TIMP1 protein in SW620 cells. The downregulation of MMP2 was observed only in combination treatment, but there was little change in Egr1 expression (Fig. 6D).

### CFZ combination of CPT-11 suppresses colorectal cancer growth in vivo

**Figure 7:**
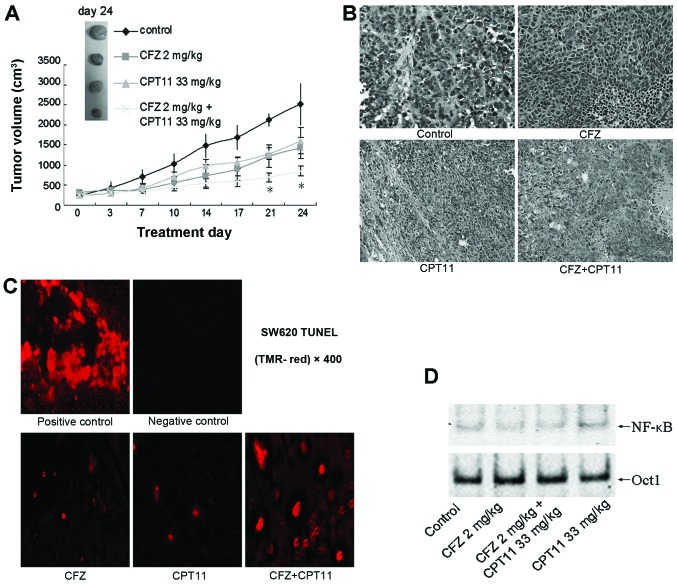
Combination effects of CFZ and CPT-11 in a SW620 xenograft model. (A) BALB/c nude mice were subcutaneously inoculated in the back with 10×10^6^ SW620 cells. Once the tumor diameter reached 7 mm, mice were treated with 2.0 mg/kg carfilzomib (i.v., twice weekly for 3 weeks) ± 33 mg/kg CPT-11 (i.p., once weekly for 4 weeks). Tumor volumes were measured twice weekly, and mean tumor volumes were plotted against days of treatment. Bars, SD. ^*^P<0.05. (B) H&E staining of tumor tissues from SW620 xenografts after indicated treatment. Magnification, ×200. (C) Representative photomicrographs demonstrating TUNEL staining of SW620 tumor sections after treatment with 2.0 mg/kg CFZ, 33 mg/kg CPT-11, and combination CFZ and CPT-11. Tumors were removed from xenografts and processed according the protocol of the In situ Cell Death Detection Kit, a fluorescent microscope was used to detect TUNEL positive (fluorescent red) cells. (D) After various indicated treatments, the tumors were excised and nuclear protein was extracted. EMSA was carried out for NF-κB activity assay. Oct-1 was used as a loading control.

To evaluate the *in vivo* anti-colorectal cancer effects of the CFZ/CPT-11 combination regimen, a SW620 cell xenograft model was used. As shown in Fig. 7A, CFZ alone or CFZ/CPT-11 co-administration suppressed tumor growth and reduced tumor size. The volume of tumors in mice receiving combination treatment was less compared with single-agent treatment, especially after day 21 (P<0.05). This regimen also significantly abrogated tumor weight; CFZ combined with CPT-11 reduced the tumor weight to 0.53±0.12 g in the final treatment, which is significant less compared with single-agent treatment groups, 1.03±0.11 g in the CFZ group and 1.22±0.04 g in the CPT-11 group (P<0.05). The H&E staining of tissue from xenografted tumors confirmed that they were malignant colorectal cancer cells (Fig. 7B). In the TUNEL staining of tissue sections, CFZ combined with CPT-11 also increased apoptosis of tumor cells (Fig. 7C). Further, excised tumor tissue assayed in the EMSA showed that CFZ alone resulted in NF-κB downregulation and combined treatment also inhibited NF-κB activation induced by CPT-11 (Fig. 7D). These findings indicate that CFZ increases the *in vivo* antitumor activity of CPT-11 in colorectal cancer by blocking NF-κB activity.

## Discussion

**Figure 8:**
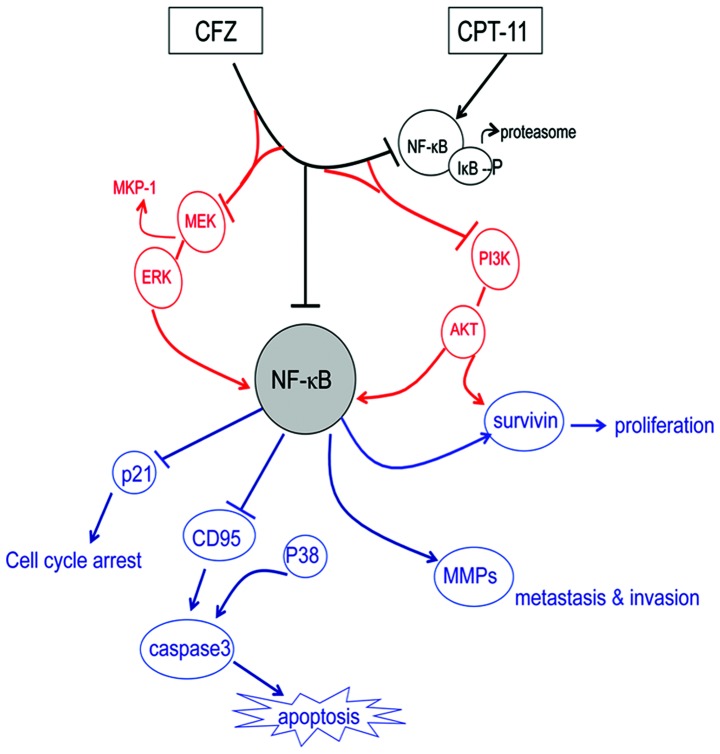
The mechanisms of the combination CFZ and CPT-11 therapeutic effects. CFZ inhibited CPT-11 induced NF-κB activation, and the combined regimen induces NF-κB inhibition related multiple pathway, including blocking the MEK/ERK pathway, PI3K/AKT pathway dephosphorylation, as well as modulating NF-κB target genes, downregulating survivin to suppress cell proliferation, increasing P21 causing cell cycle arrest, inducing apoptosis by activating CD95 and the caspase 3 pathway. Combined treatment also decreased MMP activity inhibiting cell migration and invasion.

In this study, we determined the combined therapeutic effects of carfilzomib with CPT-11 in SW620 cells and in a xenograft model, as well as investigated the mechanism of action. The results of this study indicate that CFZ significantly potentiated CPT-11 activity against SW620 cells, suppressed proliferation, inducing apoptosis, G2/M arrest, and inhibition of aggresome formation through multiple mechanisms, including NF-κB inhibition, MEK/ERK and PI3K/AKT phosphorylation modulation, survivin downregulation, and the CFZ/CPT-11 combination regimen also displays significant *in vivo* activity in a SW620 xenograft model. The schema of the proposed CFZ combination with CPT-11 is shown in Fig. 8. To the best of our knowledge, this is the first report that the second-generation proteasome inhibitor, carfilzomib, which is more selective with better effects and lower toxicity than first generation proteasome inhibitors, is an effective antitumor agent that significantly enhanced the CPT-11 chemosensitivity of colorectal cancer through inhibition of multiple NF-κB related mechanisms, and could be a potential novel therapeutic for treating colorectal cancer patients.

### CFZ interacts synergistically with CPT-11 to induce growth inhibition and apoptosis in colorectal cancer cells

Proteasome inhibitors have been used as effective antineoplastic agents, although mainly in hematologic patients (30,31). They have been shown to inhibit growth of many other solid tumors, such as ovarian carcinoma, prostate tumor and colorectal cancer (19,32–34), and have an increased anticancer potency for solid tumor treatment when combined with some conventional anticancer agents (35,36). Carfilzomib is a novel ‘second-generation’ proteasome inhibitor with a greater potency than bortezomib, and it was given an accelerated approval by the FDA and can be a safe and effective replacement for bortezomib for MM therapy (19,25,26). However, little is known about its activity against CRC. In our study, CFZ given alone induced the inhibition of growth of CRC cell lines in a concentration-dependent and time-dependent manner, and decreased the colony formation from above 400 colonies to under 100 colonies when we increased the CFZ concentration. It suppressed tumor growth along with tumor volume and tumor weights, as well as induced apoptosis both in SW620 cells and in xenografted tumors, exhibiting cytotoxic effects *in vitro* and *in vivo*. Addition of CFZ to CPT-11 greatly potentiated inhibitory effects and increased apoptosis in SW620 cells and in the xenograft model. Single-agents replaced with the combination regimen was more efficacious and the dose of CPT-11 can be reduced more than 2-fold to a less toxic dose without any loss in efficiency. The synergistic interaction was greatest between low-middle doses of CPT-11 and CFZ. Similar synergistic effects were reflected in clonogenic assays, where administration of CFZ and CPT-11 also sharply decreased colony formation of SW620 cells. The enhanced apoptosis-inducing effects of CFZ in combination with CPT-11 in SW620 cells was 1.62–2.30-fold higher compared to treatment with single agents, and the anticancer effect of the combined regimen on SW620 tumors appears to be partly due to enhanced apoptosis. These results demonstrate that CFZ not only displays a single-agent activity but also enhanced chemosensitivity to CPT-11 in CRC cells.

### CFZ applied alone or combined with CPT-11 led to G2/M arrest and further enhanced apoptosis in CRC cells

In our study, the inhibition of SW620 cell growth by combinations of CFZ with CPT-11 or by CFZ alone was accompanied by a time-dependent and concentration-dependent cell cycle arrest in G2/M. Induction of cell cycle arrest in the G2/M-phase may induce cell apoptosis if damage is extensive (37). It is noteworthy that SW620 cells exhibit abnormalities in components of DNA damage checkpoints, with reductions of cdc25c and mutant p53, which may contribute to the enhanced G2/M arrest and further the apoptotic response (38,39). As reported by McConkey and Zhu (40), in the presence of proteasome inhibition, expression of the cell cycle regulators, including cdc25a, cdc25c, KIP1, INKs and cyclins, were destabilized, which will make cells more susceptible to apoptosis. One possible explanation for this dramatic shift toward G2/M arrest was identified in the western blot analysis which showed there were decreases in cyclin D1 that regulates G1/S transition, cyclin B1/cdk1 that regulates transition in G2/M, cdc25c that functions as a cyclin B1/cdk1 phosphatase, as well as of mutant p53. There were increases in the levels of cyclin A that promote cell cycle transition from the G1 phase to the S phase and from the S phase to the G2 phase, phosphorylation of cdk1 at Thr14/Thr15, the sites associated with inhibition, and of the cell cycle negative regulator p21. Consistent with the effect on SW620 cells, it was reported that the G2/M arrest is induced along with apoptosis (41). In our study, after a moderate shift in the cell population toward G2/M arrest by addition of CFZ to CPT-11, there was a further increase of apoptosis of SW620 cells. This suggests that combining CFZ and CPT-11 induced cell cycle arrest that may play a role in mediating cell death. As shown in our results, treatment of SW620 cells with CFZ and CPT-11 resulted in the activation of caspase 3 and CD95-dependent apoptotic pathways, as well as of proteins associated with pro-apoptotic effects; this included p-p38 and ATF3 which have been shown to play an important regulatory role in the apoptosis of tumor cells (42). For these reasons, activation of caspase 3 and CD95, as well as p-p38 and ATF3, both mobilized by combined treatment, are thought to be involved in CFZ-mediated lethality and may participate in the increased apoptosis induced by CPT-11.

### Inhibition of CPT-11-induced NF-κB activation plays a significant role in the chemosensitivity of the SW620 cells

It has been demonstrated that CRC has high constitutive NF-κB expression (7). The NF-κB pathway plays a crucial role in cancer cell survival (10,43), persistent activity of NF-κB is associated with tumor formation, growth and metastasis, as well as drug resistance in many cancers (44–46). Since the proteasome pathway is important for activating NF-κB (25), the ability of a proteasome inhibitor to inhibit the NF-κB pathway by blocking proteasome degradation of IκB plays a key role in the activity of this agent against tumor cells (47–49). Zanotto-Filho *et al* reported that inhibition of the NF-κB pathway is one of the major effects by which the proteasome inhibitor induces selective apoptosis in glioblastoma cells (50). In addition, CFZ induced growth inhibition and apoptosis through inhibiting the NF-κB signaling pathways in mantle cell lymphoma (25), and co-administration abrogated NF-κB activity in vorinostat-treated granta cells and HF-4B cells (23). Moreover, synergistic anticancer activity was reported when chemotherapeutic agents such as CPT-11, were combined with NF-κB inhibitors, including proteasome inhibitors (15,18,51). Studies of NF-κB inhibition by proteasome inhibitors, such as NPI-0052, have demonstrated a synergistic response with chemotherapeutic drugs in a colon cancer model (18). Studies using the proteasome inhibitor bortezomib (PS-341), that also blocks NF-κB activation, have also demonstrated increased chemosensitivity of CRC cells to CPT-11 toxicity (16), and an increased cytotoxic effect of CPT-11 on glioma cells (52). In the study by Park *et al*, simvastatin inhibiting of the proteasome also abrogated NF-κB activation and sensitized NSCLC cells to CPT-11 induced apoptosis (53). These reports indicate that inhibition of NF-κB with proteasome inhibitors may be an important therapeutic approach to improve the effect of chemotherapy.

Consistent with these results, our findings show that CFZ is cytotoxic to human SW620 CRC cells and CFZ blocks NF-κB activation in a dose-dependent manner. We also report that combined treatment of SW620 cells with CFZ/CPT-11 will inhibit NF-κB activation that was induced by CPT-11 by blocking the degradation of IκB, and the inhibition of NF-κB was also seen when CFZ/CPT-11 was administered *in vivo*. It is possible that this is a mechanism that contributes to growth inhibition and apoptosis induced by this regimen. Moreover, we have demonstrated that SW620 cells, which have high levels of constitutively active NF-κB, as shown in the EMSA assay, are sensitive to CFZ-induced cell death, whereas HCT8 cells, that have significantly lower levels of constitutively active NF-κB, are considerably less sensitive to CFZ-induced cell death. Thus, the state of NF-κB activation correlates with CFZ sensitivity in these colorectal cancer cells. Combined with the results from EMSA and western blot analysis, this suggests that regulation of NF-κB plays an essential role in CFZ lethality.

### CFZ-enhanced anticancer effect of CPT-11 is also attributable to MEK/ERK and PI3K/AKT dephosphorylation, and survivin downregulation disrupting multiple cytoprotective signaling pathways

It is reported that proteasome inhibitors target various genes and pathways which regulate growth and survival of transformed cells; targets such as cell cycle, NF-κB, aggresome formation and stress pathways that can modulate tumor development (10). NF-κB regulation is pivotal in the therapeutic function of these proteasome inhibitors (21,50,54), it is involved in tumor progression through regulation of transcription of various genes that regulate cell proliferation, apoptosis, invasion and metastasis; including NF-κB target genes such as cyclin D1 and survivin, ERK, STATs, caspases and MMP9 (55–59). This suggests that the action of proteasome inhibitors is not limited to NF-κB inhibition but that other related mechanisms may play a role in the antitumor activity. In the report by Ye *et al*, MEK/ERK and PI3K/AKT pathways are often activated in CRC, and they cooperate to regulate survivin expression that is associated with metastatic progression and poor survival of CRC (60). Our study indicated that CFZ alone inhibited the phosphorylation of MEK, ERK, PI3K and AKT, and it decreased MKP-1 and survivin. Exposure of SW620 cells to combined CFZ and CPT-11 also attenuated the MEK/ERK pathway and reduced MKP-1, as well as inducing PI3K/AKT pathway dephosphorylation and survivin downregulation. All these effects may play a significant role in the enhanced lethality of CPT-11. These results also support the role of MKP-1 as a possible mediator between CFZ/CPT-11 treatment and induction of apoptosis. Thus, the potential of CPT-11 as an antineoplastic drug may be significantly enhanced with CFZ, not only through inhibiting NF-κB, but also by modifying factors downstream from NF-κB or survival signaling pathways dependent on proteasome function. Taken together the collective data is consistent with CFZ potentiating CPT-11 lethality by disrupting multiple cytoprotective signaling pathways.

### CFZ or combination treatment also inhibits invasion and migration of SW620 cells, with decreased MMPs and increased TIMPs

Using a wound-healing migration assay, combined with Transwell migration and Matrigel invasion assays, cell migration and invasion were found to be markedly decreased with the combination regimen, indicating that CFZ may attenuate colorectal cancer cell motility. MMPs, members of the neutral endopeptidase family that can catalyze the degradation of ECM components, are thought to play an important role in tumor invasion and metastasis in colon carcinoma and many other human cancers, and their action in tumors is inhibited by specific tissue inhibitors (TIMPs) (61,62). In our present western blot analysis results, CFZ alone or combined with CPT-11 diminished MMP1 and MMP9 protein expression, while it increased the TIMP1 protein levels in SW620 cells, which was correlated with the invasion and migration inhibition function of CFZ. The downregulation of MMP2 was observed only in combination treatment, showing that CPT-11 contributed more to these effects. This suggests that this combination regimen may act through reducing MMPs and increasing TIMPs to reduce their metastatic and invasive capability.

### Using CFZ in combination with CPT-11 might be a promising strategy for treating colorectal cancer

In summary, our data support the notion that the second-generation proteasome inhibitor CFZ should be considered a potential antitumor agent when combined with CPT-11 for the treatment of CRC. It is highly synergistic for potentiating the cytotoxic effects of CPT-11 on SW620 colorectal cancer cells and in a tumor xenograft model. It actively mediates multiple mechanisms, including NF-κB inhibition, MEK/ERK pathway blocking, PI3K/AKT pathway dephosphorylation, and survivin downregulation, and these events are accompanied by cell cycle arrest, increased apoptosis, as well as inhibition of cell migration and invasion. Thus, this regimen warrants consideration as a therapeutic strategy for CRC and merits further clinical studies.

## Figures and Tables

**Table I:** The combined effects of CFZ and CPT-11 on SW620 cells (mean ± SEM, n=3).

CFZ (nM)	CPT-11 (μM)	Inhibition rate (%)	CI-value
0	0	0	
	50	26.87±3.28	
	100	48.19±1.84	
	200	71.63±2.91	
10	0	10.62±2.78	
	50	52.29±3.17	0.64
	100	71.26±2.76	0.60
	200	75.85±5.92	0.93
20	0	28.16±3.75	
	50	59.03±2.33	0.67
	100	85.39±8.11	0.37
	200	75.54±5.93	1.02
50	0	54.09±1.55	
	50	75.94±2.57	0.61
	100	84.25±2.56	0.55
	200	91.63±6.52	0.49
100	0	66.98±1.99	
	50	80.95±5.79	0.79
	100	89.23±1.90	0.57
	200	86.88±2.31	0.93
